# Outcome Report of Treatment for Sexual Offenders in Japan

**DOI:** 10.5964/sotrap.12581

**Published:** 2026-08-18

**Authors:** Atsushi Nishida, Eiji Saito

**Affiliations:** 1 Kawagoe Juvenile Prison, Ministry of Justice, Saitama, Japan; 2 Centre for Evidence-Based Research, Ministry of Justice, Tokyo, Japan; Simon Fraser University, Burnaby, Canada; University Medical Center Mainz, Mainz, Germany

**Keywords:** Specialized Sex Offender Treatment Programme (SSOTP), sex offender, outcome study, sexual recidivism, Japan

## Abstract

This study examined the effectiveness of Specialised Sex Offender Treatment Programmes (SSOTP) provided at correctional facilities in Japan. Because sexual offences are serious crimes that invoke public concern, providing effective treatment for sex offenders is crucial. However, there is insufficient evidence on the effectiveness of this treatment for sex offenders, particularly in Japan. Therefore, this study investigated whether receiving SSOTP reduced general and sexual recidivism. All participants were followed up for three years from the day when they were released from correctional facilities in Japan. The results revealed that the rate of sexual recidivism in participants who received SSOTP (15.0%, *n* = 1,444) was significantly lower than that of those who did not (22,5%, *n* = 324). Receiving SSOTP was also associated with reduction in general recidivism (treated offenders: 27.3%; untreated offenders: 38.0%). Further research utilizing more robust methodologies is warranted for more rigorous corroboration.

## Highlights

- Second evaluation study on SSOTP provided at correctional facilities in Japan was conducted.
- Receiving SSOTP was correlated with a reduction in both general and sexual recidivism.
- More rigorous research is warranted to establish more robust efficacy of SSOTP.

Sexual offences are serious crimes that invoke public concern. Once a sexual offence occurs, victims experience serious physical and mental damage. Dworkin et al. (2017) observe that sexual assault victimisation is associated with increased risk for multiple psychopathologies such as depression and post-traumatic stress disorder (PTSD). Farrington and Welsh (2023) showed that rehabilitating offenders incurs enormous financial and social costs and explained the importance of measuring all financial costs of crime. From a cost-benefit perspective, each sexual offence costs £19,000 which is more expensive than other crimes such as theft (Brand & Price, 2000). The cost of sexual offences has increased in recent years, and each rape costs £39,360 which is the highest estimated unit cost of non-fatal offences (Heeks et al., 2018). In addition, sexual offences tend to be underreported, resulting in a significant underestimation of their actual prevalence. Therefore, there is a high demand for the treatment of sexual offenders. Given the global efforts to provide and evaluate sex offender treatment for recidivism prevention, the implementation of evidence-based interventions remains a critical priority.

Evaluation studies on sex offender treatment have been conducted in many countries, especially in North America, but their results remain controversial (Lösel & Schmucker, 2005; Schmucker & Lösel, 2015, 2017). Hanson et al. (2009) examined whether the Risk-Need-Responsivity (RNR) principles (Andrews & Bonta, 2010)—which play a central role in treating general offender groups—can be applied to sex offender treatment. Schmucker and Lösel (2017) conducted a large-scale meta-analysis and noted that sex offender treatment for reducing recidivism is effective; however, the results of individual studies are not homogenous, and more research which adopts randomised control trials (RCT) or good quasi-experimental design is required. In particular, it remains unclear whether the programmes provided in prison settings are effective in mitigating recidivism.

In Japan, structured sex offender treatment programmes known as Specialised Sex Offender Treatment Programmes (SSOTP) were introduced in penal institutions in 2006. In response to growing public concern and calls for more systemic and effective management of sexual offenders—such as a released inmate who had kidnapped and murdered a girl in 2004—and against the background of the enforcement of the Act on Penal Detention Facilities and Treatment of Inmates and Detainees in 2006, the Japanese Correctional Bureau implemented comprehensive treatment programmes based on previous efforts and research in Western countries, especially Canada, with sophisticated, evidence-based offender treatment systems. As a basis of treatment, cognitive behavioural group therapy was adopted because it was regarded as an optimum choice from the perspectives of its effectiveness and cost performance.

The prevention of sexual offences has attracted public concern in Japan. According to recent government statistics (White Paper on Crime, 2024), the number of sexual violence incidents in Japan in 2021 was 5,671. The prevalence rate of sexual violence was 4.6% and it remains around 5.0% over the last five years. Although this rate is lower than that of other countries such as South Korea, France, and Germany, the recidivism rate of sex offenders who committed penetrative sexual assault or indecent assault and were released from penal institutions is 17.1%, which is almost the same as the rates of robbery (18.8%) or fraud (20.5%) (White Paper on Crime, 2024). In addition, a contact sexual offence in the crowded train called ‘chikan’ in Japanese is a serious problem in Japan. According to the Cabinet Office of Japan (2022), more than one in ten girls and women (10.3%) has a history of victimisation of train groping. Typical train gropers had histories of several criminal charges, which were primarily the same offence type, with others being compulsive acts of repeated indecency (White Paper on Crime, 2014). Therefore, effective management of sexual offenders and prevention of recidivism have become imperative not only for policy makers but also for the public, and consequently, several policies regarding sexual offences have been implemented.

Considering the circumstances stated above, evidence for the effectiveness of sex offender treatment in Japan is neither clear nor sufficient. In 2012, the first evaluation study of SSOTP was conducted by the Japanese Ministry of Justice. The study aimed to clarify the treatment effect of the SSOTP by comparing the recidivism of treated sex offenders with that of untreated sex offenders in Japanese penal institutions. A significant reduction in general recidivism rates in the treated group was observed, suggesting that SSOTP prevented general recidivism. However, the effectiveness of these programmes on sexual recidivism remains limited, owing to methodological limitations such as inadequate follow-up. Taken together, revision of SSOTP, and training programme facilitators were recommended to enhance treatment effectiveness, and accordingly, further research was suggested.

In response to the first evaluation study in 2012, the Correctional Bureau of the Japanese Ministry of Justice launched a preparation programme. The preparation program which aims to improve the motivation of SSOTP participants works as a booster. Because the programme is mandatory and affected by the characteristics of sex offenders, participants often lack motivation for treatment, and facilitators face challenges in enrolling them into SSOTP. Therefore, following Miller and Rollnick (1991), the preparation programme consists of four sessions to enhance motivation for enrolment.

Concurrently with the implementation of the preparation programme, the upskilling of facilitators was promoted by the quality supervision of external experts, intensive training at induction, and ongoing training to deal with the various complex problems faced by sex offenders and provide more effective intervention programmes.

In sum, evidence regarding the effectiveness of sex offender treatment in Japan remains scarce and verification of the revisions of SSOTP that address issues identified in the first evaluation from a program evaluation point of view is essential.

## The Present Study

The present study aimed to examine the effectiveness of sex offender treatment programmes provided in Japanese penal institutions by comparing the recidivism rates of those who had received SSOTP intervention with those who had not. As noted above, while the efficacy of sex offender treatment programmes has been sufficiently established, the results of evaluation studies are heterogeneous (Schmucker & Lösel, 2015, 2017). Considering that many outcome studies have been conducted in Western countries—North America in particular—this study aimed to examine the effectiveness of sex offender treatment in Japan and contribute to the discussion on tangible outcomes for sex offenders.

### Assessment of Sex Offenders in Penal Institutions in Japan

The assessment of sex offenders in penal institutions in Japan is based on Risk-Need-Responsivity principles (Andrews & Bonta, 2010). All new male inmates in penal institutions are screened at the beginning of their sentences to determine whether they have been thoroughly assessed and treated as candidates for SSOTP^1^1Sex offenders who commit at least one contact offense are eligible for detailed assessment. In other words, sex offenders who commit non-contact offenses only are excluded for detailed risk-need assessment.. When they meet the screening criteria, psychological staff assess their recidivism risk and criminogenic needs using the Risk Assessment Tool (RAT) and Needs Assessment Tool (NAT) which were developed by the Japanese Ministry of Justice. Based on these assessments, treatment intensity, namely low, moderate, or high intensity, is determined. In addition to risk and needs assessment, the responsivity issues of sex offenders, such as intellectual disabilities, mental or physical problems, and motivation for treatment, are also considered. For example, individuals with intellectual disabilities are assigned to an Adapted Program which addresses their responsivity factors. Finally, their treatment plans are settled with reference to their sentence duration, timing of programme implementation, etc. The results of the assessment and treatment plan for sex offenders are registered in the database owned by the Correctional Bureau of the Japanese Ministry of Justice. Based on this information, the regional and central headquarters of the Correctional Bureau centrally ensure that all the eligible sex offenders receive SSOTP. Once sex offenders in the Japanese penal institution are designated to receive SSOTP, entering the programme is mandatory, except for special reasons such as physical or mental issues and sentence durations that are too short. Candidates with long sentences wait for treatment in penal facilities which do not provide SSOTP and are transferred to the institution providing SSOTP at certain periods of their sentences.

### Treatment of Sex Offenders in Penal Institutions in Japan

The design of SSOTP was derived from a thorough review of the sex offender treatment literature and the input of experts from Canada. The programme includes the following steps: Orientation, (Preparation Programme), Primary Treatment Programme, and Maintenance Programme.

**Figure 1:**
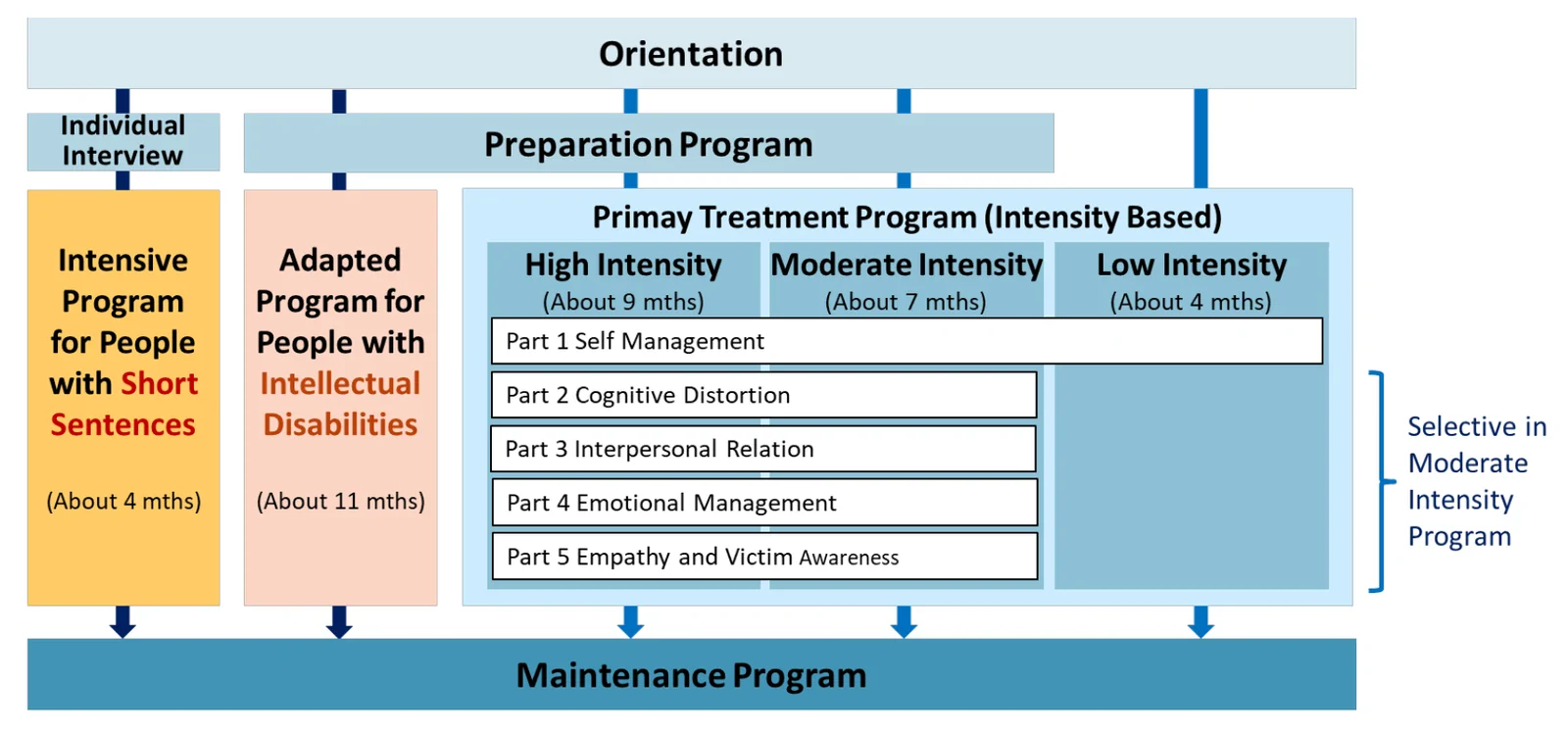
Components of Specialized Sex Offender Treatment Programmes (SSOTP)

The participants in SSOTP are male inmates with problems related to sex crimes, such as distorted cognition and a lack of self-control. Female sex offenders are not eligible for SSOTP. Treatment goals are to understand the specific problems that drive them to commit sexual crimes, work towards eliminating those problems, and acquire knowledge and skills to prevent recidivism. The types of treatment programmes are presented in Figure 1.

Participants were assigned to high-, moderate-, or low-intensity programmes based on their risks and needs. The high-intensity programme requires participants to address all five components over nine months. The moderate-intensity programme requires participants to address Part 1 and other parts depending on their necessities for recidivism prevention over approximately seven months. The low-intensity programme requires participants to address Part 1 for only approximately four months due to their low risk of recidivism. As stated above, considering the characteristics of sex offenders, a preparation programme was developed to enhance their low motivation and readiness for treatment based on Miller and Rollnick (1991). The preparation programme is provided before the high-intensity, moderate-intensity, and adapted programs and works as a booster.

Each treatment group that is provided in a fixed format, consisted of approximately eight participants and two or three facilitators. SSOTP are typically conducted in groups, but depending on necessity, supplemental individual sessions are provided. Programme facilitators comprise psychologists, educational specialists, or prison officials in penal institutions trained in SSOTP delivery.

In addition to the abovementioned mainstream sex offender treatment programmes, two other programmes based on their responsivity are conducted, such as an adapted programme, designed for individuals with intellectual disabilities. The programme was derived from Haaven et al. (1990) and involves the incorporation of visual information, a slower pace of treatment, and repetition of treatment elements tailored to their intellectual abilities and to enhance treatment gains as optimally as possible. Based on the RNR principles, low-risk sex offenders are not eligible for the adapted programme. Another additional programme is an intensive program for those who do not have sufficient sentence durations to complete the primary programme. The components of the intensive programme are a shortened version of Part 1 of the primary programme which is intended to improve self-management.

## Method

### Participants

We used national data on 1,768 SSOTP candidates released from penal institutions between 2012 and 2014. Of the 1,768 candidates, 1,444 participants who had completed SSOTP were defined as the treatment group (TG), and 324 participants who had not received SSOTP were defined as the comparison group (CG). Participants who dropped out of SSOTP were included in the treatment group regardless of their attendance rate. Participants in the comparison group were those who needed but could not receive SSOTP for reasons such as insufficient sentence duration. Those who had not received the SSOTP due to physical or mental problems were considered inappropriate for comparison and excluded from comparison group to preserve the equality of both groups.

### Risk Assessment Tool 2005 (RAT 2005)

The Risk Assessment Tool (RAT), developed by the Correctional Bureau of the Japanese Ministry of Justice in 2005, has been implemented to assess the recidivism risk of sexual offenders detained in penal facilities in Japan. The RAT 2005 is applicable for male sex offenders over 18 years of age who have a prior conviction of sex offences. In the course of its development, the RAT 2005 was based on the risk principle, with reference to the risk assessment tools Static-99 (Hanson & Thornton, 1999), The Sex Offender Risk Appraisal Guide (SORAG) (Quinsey et al., 1998), and Minnesota Sex Offender Screening Tool Revised (MnSOST-R) (Epperson et al., 2003).

The RAT 2005 is scored by psychological staff at penal institutions and consists of three domains and 36 items^2^2There are some duplicated items in RAT-1, RAT-2, and RAT-3.. Three domains were named RAT-1 (10 items), RAT-2 (10 items), and RAT-3 (16 items). RAT-1 is used for risk assessment and is based on the selection of the programme intensity. RAT-2 and RAT-3 are used as references, and also aim to collect data for examining its validity and future standardisation. This study used RAT-1 as an index of the static risk of the participants (RAT 2005 score). The RAT-1 consists of 10 items, including history of previous sexual offences and history of noncontact sex offences. Nine of ten items are scored dichotomously, namely 0 or 1, and only one item is scored from 0-3. The scoring criteria depend on each item. The total RAT score is the sum of all items. The RAT score ranges from 0–12, and four risk levels, low, low-moderate, moderate-high, and high, are categorised by their total scores. Their level is used for selecting treatment intensity with reference to the NAT level explained below. Higher scores indicate a higher risk of recidivism.

The RAT was revised three times by 2023 to correspond to the amendment of penal codes or to add variables for program evaluation. Although the newest version is RAT 2023, this study used RAT 2005 because of the time of assessment of participants and evaluation of the tool.

### Needs Assessment Tool 2005 (NAT 2005)

The Needs Assessment Tool (NAT) was developed by the Correctional Bureau of the Japanese Ministry of Justice in 2005 and has been implemented to assess the criminogenic needs of sexual offenders detained in penal facilities in Japan. In the course of its development, NAT 2005 was based on the need principle, with reference to STABLE-2000 (Hanson & Harris, 2001).

NAT 2005 consists of six domains such as Intimacy Problems, Lack of Self-Control, and 16 items such as Hostility toward Women, and Deviant Sexual Interests. According to the scoring manual, psychological staff at the assessment centres in eight Japanese penal institutions score each item through semi-structured interviews and file reviews as a part of a detailed assessment at the beginning of participants’ sentences to classify inmates based on their criminal tendency, sentence duration, mental and physical condition, etc. Each item is rated from 0 (not true) – 2 (true). Four out of six domains consist of several items, and the item with the highest score in a domain is regarded as the domain score. Finally, the NAT score is calculated by summing up all domain scores. The NAT score ranges from 0 – 12, and is classified into three need levels: low, medium, and high. The need level is used for selecting treatment intensity based on the RAT level.

### Definition of Recidivism

In the current study, we refer to general (any) recidivism as the earliest criminal case prosecuted by a prosecutor within three years after the candidate’s release from penal institutions, and sexual recidivism as the earliest sex offence case prosecuted by a prosecutor within the same period^3^3Sexual recidivism included the following names of offenses, rape indecency through compulsion, offense for the purpose of indecency, Against Nuisance Prevention Order, Violation of The Child Welfare Act/ Ordinance Regarding the healthy development of Youth which are the same categorization of participants’ index offenses shown in Table 1.; therefore, the observation period for all participants was three years. In other words, we started to follow the participants from the time they were released from penal institutions until the time they committed a general (sexual) offence prosecuted by a prosecutor, or otherwise, 1,095 days after their release from penal institutions. Breaches were not included in recidivism. Data on recidivism were collected from official records and the database managed by the Japanese Ministry of Justice.

### Measures and Analytic Strategy

We first used an independent sample *t*-test to compare the mean of demographic data such as the number of incarcerations, sentence duration, age at release, RAT total score, and NAT total score between the treatment group (TG) and the comparison group (CG). We then used a chi-square test to compare the recidivism rate and distribution of the names of charges between the TG and CG.

Second, we verified whether the RAT score accurately measured recidivism risk. Logistic regression analyses showed that the RAT score was an independent variable and general or sexual recidivism was a dependent variable. The area under the receiver operating characteristic (ROC) curve (AUC) was then calculated.

Finally, to examine treatment effects, the Cox Proportional Hazard Model was used. General recidivism or sexual recidivism was the dependent variable, the observation period was the survival variable, and whether participants received treatment (1 = treatment group, 0 = comparison group) and the RAT score were co-variables.

## Results

### Descriptive Statistics

#### Distribution of Participants by Offence

Table 1 presents the distribution of participants by offence.

**Table 1:** Distribution of Participants by Offence

Sexual offence category	Treatment group (*n* = 1,444)		Comparison group (*n* = 324)		Total (*N* = 1,768)	Component ratio	χ^2^	Cramer’s V
	*n*	Component ratio	*n*	Component ratio				
Rape	480	33.2	73	22.5	553	31.3	186.18**	0.32
Indecency through Compulsion	653	45.2	92	28.4	745	42.1		
Offensed for the purpose of indecency	3	0.2	1	0.3	4	0.2		
Against Nuisance Prevention Ordinance	81	5.6	82	25.3	163	9.2		
Violation of the Child Welfare Act/ Ordinance Regarding the Healthy Development of Youths	176	12.2	36	11.1	212	12.0		
Violent Crimes other than sex offenses	22	1.5	9	2.8	31	1.8		
Others	29	2	31	9.6	60	3.4		

**p < .01.

A Chi-square test showed significant difference among conditions (χ^2^(6) = 186.18, *p* < .01, Cramer’s V = 0.32). Residual analysis revealed that the TG significantly included more incidences of rape and indecency through compulsion than the CG, and the CG significantly included more incidences of Against Nuisance Prevention Ordinance and Others than the TG.

#### Descriptive Statistics of the Participants

Table 2 presents the participants’ descriptive statistics.

**Table 2:** Descriptive Statistics of the Participants

Variable	Treatment group (*N* = 1,444)		Comparison group (*N* = 324)		*t*	Effect size *d*
	*M*	*SD*	*M*	*SD*		
Number of incarcerations	1.77	1.65	2.11	2.04	2.77**	0.19
Length of stay in penal institutions (days)	1290.43	725.76	1107.18	1369.39	2.34*	0.21
Length of sentences (Months)	46.07	24.82	37.03	41.89	3.74*	0.31
Age at release	39.59	11.19	43.31	13.33	4.67*	0.32
RAT2005 score (0-12)	3.99	2.05	4.53	1.90	4.36*	0.27
NAT2005 score (0-12)	7.07	1.83	7.33	1.92	2.31*	0.14
Intellectual abilities	89.44	13.43	86.89	15.84	2.67*	0.18

*Note.* Due to the missing values of Intellectual abilities, *N* of TG is 1,440 and *N* of CG is 318.

*p < .05. **p < .01.

All items showed significant differences between the TG and CGs. The TG had fewer incarcerations, longer lengths of stay in penal institutions and sentences, a younger age at release, lower RAT and NAT scores, and higher intellectual abilities than the CG.

#### Composition of Participants in the TG Based on the Type of Treatment Programme

Table 3 presents the composition of the participants in the TG according to the type of treatment programme.

**Table 3:** Composition of Participants in the TG Based on the Type of Treatment Programme

Type of Programme	*N*	Component ratio (%)
High Intensity	350	24.2
Moderate Intensity	747	51.7
Low Intensity	192	13.3
Intensive Programme	57	3.9
Adapted Programme	98	6.8
Total	1444	100.0

*Note.* Sum of the component ratio is not 100.0% because of the round off of the second decimal place.

Participants received Moderate intensity programmes the most (51.7%), followed by High intensity (24.2%), Low intensity (13.3%), Adapted (6.8%), and Intensive programmes (3.9%).

#### Composition of the Participants Based on Offence Type

Table 4 presents the composition of the participants according to the type of offence.

**Table 4:** Composition of the Participants According to the Type of Offense and Treatment Programme

Sexual offense category	High intensity (*N* = 350)			Moderate intensity (*N* = 747)			Low intensity (*N* = 192)			Intensive program (*N* = 57)			Adapted program (*N* = 98)			Total (*N* = 1,768)	CR
	*N*	CR in the program	CR in the offense type	*N*	CR in the program	CR in the offense type	*N*	CR in the program	CR in the offense type	*N*	CR in the program	CR in the offense type	*N*	CR in the program	CR in the offense type		
Rape	114	32.6	23.8	285	38.2	59.4	62	32.3	12.9	1	1.8	0.2	18	18.4	3.8	480	33.2
Indecency through compulsion	173	49.4	26.5	352	47.1	53.9	56	29.2	8.6	9	15.8	1.4	63	64.3	9.6	653	45.2
Offensed for the purpose of indecency	2	0.6	66.7	1	0.1	33.3	0	0.0	0.0	0	0.0	0.0	0	0.0	0.0	3	0.5
Against Nuisance Prevention Ordinance	30	8.6	37	11	1.5	13.6	0	0.0	0.0	334	59.6	42	6	6.1	7.4	81	5.6
Violation of the Child Welfare Act/Ordinance Regarding the Healthy Development of Youths	16	4.6	9.1	77	10.3	43.8	73	38	41.5	6	10.5	3.4	4	4.1	2.3	176	12.2
Violent crimes other than sex offenses	9	2.6	40.9	8	1.1	36.4	0	0.0	0.0	3	5.3	13.6	2	2	9.1	22	1.5
Others	6	1.7	20.7	13	1.7	44.8	1	0.5	3.4	4	7	13.8	5	5.1	17.2	29	2

*Note.* CR = Component ratio.

The most common type of offence was Indecency through Compulsion in high-, moderate-, and adapted programmes. Conversely, the Violation of Child Welfare Act/Ordinance Regarding the Healthy Development of Youth was the highest number of participants in the low-intensity and Against Nuisance Prevention Ordinance in the intensive programme.

### Overall Analysis of All the Participants

#### Analysis Regarding Recidivism Rates

Of the 1,768 participants, those who committed general recidivism during the observation period were 517 at a recidivism rate of 29.2%. Those who committed sexual recidivism during the observation period were 290 with a recidivism rate of 16.4%.

Table 5 presents a comparison of recidivism between the TG and CG.

**Table 5:** The Comparisons of General and Sexual Recidivism Rates

Recidivism category	Treatment group (*N* = 1,444)		Comparison group (*N* = 324)		χ^2^	φ
	Recidivist	Non-recidivist	Recidivist	Non-recidivist		
General offense	394 (27.3%)	1050 (72.7%)	123 (38.0%)	201 (62.0%)	14.58**	0.091
Sexual offense	217 (15.0%)	1227 (85.0%)	73 (22.5%)	251 (77.5%)	10.87**	0.078

*Note.* (%) indicates component ratios in each group.

**p < .01.

The differences in recidivism rates of general and sexual offences between the TG and CG were statistically significant, and the rates of the TG were lower than those of the CG. As shown in Tables 1 and 2, the variables affecting the recidivism rate, such as the component ratio of the offence type and recidivism risk assessed by the RAT, were significantly different between the TG and CG. This implies that the groups were not equivalent, and merely comparing the recidivism rates of the TG and CG was inappropriate. Therefore, by referring to the evaluation study by the Correctional Service of Canada (2008), we first controlled for the risk of recidivism in both groups using the Cox Proportional Hazard model and then examined the relationship between the SSOTP and recidivism.

#### Examination of Treatment Effectiveness by Controlling Recidivism Risk

##### Preparatory Analysis of the Control Variable

The AUC score of general recidivism was 0.72 (95% confidence intervals (CI) 0.69-0.74) and that of sexual recidivism was 0.71 (95% CI [0.68, 0.74]). The AUC scores were fair, and the RAT score predicted the risk of recidivism. Hence, the predictive validity of the RAT scores was verified.

##### The Effectiveness of the Treatment for General Recidivism

The Cox Proportional Hazard Model was used to examine treatment effectiveness for general recidivism. Table 6 presents the results.

**Table 6:** The Results of the Cox Proportional Hazard Model for General Recidivism

Covariates	Coefficient	Wald	Hazard ratio	95% CI	
TG/CG	-0.24	5.36*	0.79	0.64	0.96
RAT score	0.32	222.94*	1.38	1.32	1.44

*Note. N* of participants = 1,768; *N* of any type of recidivist = 517.

*p < .05.

A statistically significant difference was found (Hazard ratio = 0.79, 95% CI [0.64, 0.96]) between the TG and CG with respect to general recidivism. This suggests that SSOTP relates to a reduction in general recidivism, and after controlling for recidivism risk, the TG was less likely than the CG to commit a general re-offence within three years from their release.

##### Treatment Effectiveness for Sexual Recidivism

The Cox Proportional Hazards Model was used to examine the effectiveness of the treatment for sexual recidivism. Table 7 presents the results.

**Table 7:** The Results of the Cox Proportional Hazards Model for Sexual Recidivism

Covariates	Coefficient	Wald	Hazard ratio	95% CI	
TG/CG	-0.29	4.70*	0.75	0.57	0.97
RAT score	0.32	125.31**	1.38	1.30	1.44

*Note. N* of participants = 1,768; *N* of any type of recidivist = 290.

*p < .05. **p < .01.

A statistically significant difference was found (Hazard Ratio = 0.75, 95% CI [0.57, 0.97]) between the TG and CG with respect to sexual recidivism. This suggests that SSOTP is related to the reduction of sexual recidivism, and after controlling for recidivism risk, the TG was less likely to commit sexual re-offense within three years of their release than the CG.

## Discussion

The hypothesis that receiving SSOTP reduces general and sexual recidivism was supported. This implies that SSOTP is provided in accordance with the aim of treating and mitigating sexual recidivism. This result is consistent with a meta-analysis of sex offender treatments conducted by Hanson et al. (2009), which showed that the general and sexual recidivism rates of treated sex offenders were significantly lower than those of untreated sex offenders. This also coincides with a large-scale meta-analysis by Lösel and Schmucker (2005); Schmucker and Lösel (2015, 2017), who also pointed out that a sex offender treatment programme reduced general and sexual recidivism. This is likely because the Correctional Bureau of the Japanese Ministry of Justice has been enriching the programmes and raising the number of facilitators since SSOTP was launched, and the association between SSOTP and the reduction of recidivism was sufficiently verified. In other words, compared to the first evaluation research which suggested issues related to programme fidelity (Ministry of Justice, 2012), facilitators of SSOTP had been more skilled and experienced, and treatment fidelity increased; hence, such efforts resulted in improvement of treatment quality and effectiveness. In addition, the reduction of general recidivism on receiving SSOTP indicate that the programmes are based on cognitive-behavioural therapy and relapse prevention which are applied not only to sex offenders but also to other types of offenders, and have been proven to facilitate participants’ understanding of their own problems and learning self-control (Takahashi, 2006).

### Limitations

This study is based on national data on sex offenders in Japanese penal facilities. Owing to the nature of the data and the research design, it is important to consider the following limitations when interpreting the results.

First, the proportion of sexual offences differed between the TG and CG. Due to ethical reasons and the mandatory basis of program participation, we could not conduct randomised control trials (RCT). Participants in the CG were selected from among those who should have received SSOTP, and those who were not suitable for comparison were excluded. Most participants in the CG opposed the Nuisance Prevention Ordinance and did not face sufficient sentence durations to receive even the intensive program. As stated above, sex offenders are heterogeneous in terms of mechanisms, functions, and recidivism rates. Although recidivism risk was controlled for by the RAT score, the proportion of sexual offences in the TG and CG differed when interpreting the results.

Second, the analysis by treatment intensity requires further investigation. Schmucker and Lösel (2015) note that the treatment effect depends on the risk level of sex offenders, and medium- and high-risk sex offenders typically benefit from treatment programmes, whereas low-risk sex offenders do not. This suggests that SSOTP work differently at the risk-need level, and further research is required to establish greater efficacy.

Third, the type of sexual offence should also be considered. This study examined the effectiveness of SSOTP for all participants as a first step in programme evaluation, but did not classify them based on offence type. Since sexual offending is highly heterogeneous (Schmucker & Lösel, 2017) and there is a significant body of research on sex offender typologies (e.g. Simons, 2015), the effectiveness of SSOTP may depend on offence type, and identifying their respective characteristics of risk and need is essential.

Fourth, the short-term outcomes should be considered in future studies. The present study examined the effectiveness of SSOTP in preventing recidivism. Although the public is especially concerned whether SSOTP reduces recidivism rates of sex offenders and many evaluation studies evaluate recidivism as an outcome, short-term outcomes such as reduction of dynamic risk (criminogenic need) or changes in psychometric tests are important from the perspectives of case management and programme evaluation. As Marques et al. (2005) observe, the recidivism rates of those who demonstrate an understanding of the important concepts of the program are lower than those who do not. Therefore, short-term outcomes should be considered when evaluating programme effectiveness more precisely.

Finally, confounding factors affecting recidivism should be considered. Some participants in the TG and CG were released on parole, whereas others were not. In the Japanese judicial system, those who were released on parole receive supervision from probation officers, and some of them attended community-based treatment programmes for sex offenders provided by the Rehabilitation Bureau of the Japanese Ministry of Justice. These interventions are interpreted as resources to prevent recidivism; therefore, such variables might have affected participants’ recidivism.

### Conclusion

This study aimed to verify the effectiveness of sex offender treatment programmes provided by Japanese penal institutions. The results revealed that SSOTP was correlated with a reduction in both general and sexual recidivism, which is consistent with previous studies (Hanson et al., 2009; Lösel & Schmucker, 2005; Schmucker & Lösel, 2015, 2017). However, the present study was the first step in the evaluation of the programme, focusing on sex offenders overall, and more rigorous research is warranted to establish more robust efficacy of these programmes. In addition, as SSOTP have been revised in line with the results of the present study and the literature on sex offender treatment in foreign countries, it is important to continuously evaluate the effectiveness of interventions for sex offenders, which would contribute to more effective recidivism prevention interventions, and result in a safer and healthier society for all citizens alike.

## Data Availability

The data that support the findings of this study are available from Japanese Ministry of Justice but restrictions apply to the availability of these data, so they are not publicly available. The Correction Bureau of Japanese Ministry of Justice collected and owns the data. We got a permission to use these data for our study from the Correction Bureau of Japanese Ministry of Justice.
